# Androgen promotes squamous differentiation of atypical cells in cervical intraepithelial neoplasia via an ELF3‐dependent pathway

**DOI:** 10.1002/cam4.5824

**Published:** 2023-03-23

**Authors:** Takeo Matsumoto, Takuma Suzuki, Mitsuhiro Nakamura, Megumi Yamamoto, Takashi Iizuka, Masanori Ono, Kyosuke Kagami, Haruki Kasama, Tatsuhito Kanda, Yuya Sakai, Junpei Iwadare, Ayumi Matsuoka, Kayo Kayahashi, Kousho Wakae, Masamichi Muramatsu, Satoru Kyo, Yasuhiko Yamamoto, Yasunari Mizumoto, Takiko Daikoku, Hiroshi Fujiwara

**Affiliations:** ^1^ Department of Obstetrics and Gynecology, Graduate School of Medical Sciences Kanazawa University Kanazawa Ishikawa 920‐8641 Japan; ^2^ Department of Obstetrics and Gynecology, Public Central Hospital of Matto Ishikawa Hakusan Japan; ^3^ Department of Obstetrics and Gynecology Tokyo Medical University Tokyo 160‐0023 Japan; ^4^ Department of Virology II National Institute of Infectious Diseases Tokyo 162‐8640 Japan; ^5^ Department of Obstetrics and Gynecology Shimane University Faculty of Medicine Izumo Shimane 693‐8501 Japan; ^6^ Departments of Biochemistry and Molecular Vascular Biology Kanazawa University Graduate School of Medical Sciences Kanazawa Ishikawa 920‐8640 Japan; ^7^ Division of Animal Disease Model, Research Center for Experimental Modeling of Human Disease Kanazawa University Kanazawa Ishikawa 920‐8640 Japan

**Keywords:** androgen, cervical intraepithelial neoplasia, ELF3, squamous differentiation, W12

## Abstract

**Background:**

Since the human papillomavirus vaccines do not eliminate preexisting infections, nonsurgical alternative approaches to cervical intraepithelial neoplasia (CIN) have been required. We previously reported that FOXP4 (forkhead box transcription factor P4) promoted proliferation and inhibited squamous differentiation of CIN1‐derived W12 cells. Since it was reported that FOXP expressions were regulated by the androgen/androgen receptor (AR) complex and AR was expressed on the CIN lesions, in this study we examined the effects of androgen on CIN progression.

**Methods:**

Since AR expression was negative in W12 cells and HaCaT cells, a human male skin‐derived keratinocyte cell line, we transfected AR to these cell lines and investigated the effects of dihydrotestosterone (DHT) on their proliferation and squamous differentiation. We also examined the immunohistochemical expression of AR in CIN lesions.

**Results:**

DHT reduced the intranuclear expression of FOXP4, attenuating cell proliferation and promoting squamous differentiation in AR‐transfected W12 cells. Si‐RNA treatments showed that DHT induced the expression of squamous differentiation‐related genes in AR‐transfected W12 cells via an ELF3‐dependent pathway. DHT also reduced FOXP4 expression in AR‐transfected HaCaT cells. An immunohistochemical study showed that AR was expressed in the basal to parabasal layers of the normal cervical epithelium. In CIN1 and 2 lesions, AR was detected in atypical squamous cells, whereas AR expression had almost disappeared in the CIN3 lesion and was not detected in SCC, suggesting that androgens do not act to promote squamous differentiation in the late stages of CIN.

**Conclusion:**

Androgen is a novel factor that regulates squamous differentiation in the early stage of CIN, providing a new strategy for nonsurgical and hormone‐induced differentiation therapy against CIN1 and CIN2.

## Graphical abstract

Androgen inhibits cell proliferation and promotes the differentiation of atypical cells in CIN lesions via an ELF3‐dependent pathway partially by attenuating FOXP4 function. This indicates that androgen is a possible therapeutic agent for non‐surgical CIN treatment that inhibits CIN progression by inducing squamous differentiation.

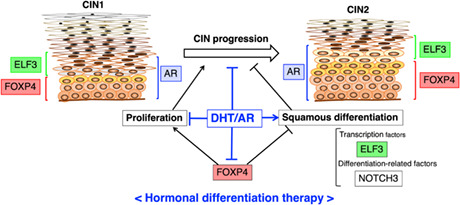

## 1 INTRODUCTION

It is widely known that human papillomavirus (HPV) is the main cause of the onset and development of CIN and cervical squamous cell carcinoma (SCC).^1^ Currently, prophylactic HPV vaccines have been demonstrated to be effective in the prevention of cervical cancers in the world.^2^ However, since these vaccines do not eliminate preexisting infections,^3^ the discovery of a new strategy to regulate HPV‐associated oncogenesis is warranted.^3^, ^4^

The initial pathological sign for CIN is impairment of squamous differentiation, which develops atypical squamous cell layers.^5^ It was reported that squamous differentiation was inhibited by HPV16 oncoproteins, E6 and E7, via MAML1, NOTCH, and PTPN14.^6^, ^7^, ^8^, ^9^, ^10^ However, since the production of E6 and E7 becomes dominant after DNA integration of HPV genes at the later stages of CIN, the main factor that impairs squamous differentiation at the early stages is still unknown.

Recently, we found that FOXP4 was expressed in atypical squamous cells in CIN.^11^ The FOXP family (FOXP1–4) was reported to regulate tissue development or cell differentiation.^12^, ^13^ In CIN1‐derived HPV16‐positive W12 cells, the downregulation of FOXP4 attenuated cell proliferation, whereas this treatment induced squamous differentiation. This FOXP4 downregulation‐induced squamous differentiation was regulated through an ELF3‐dependent pathway, suggesting that FOXP4 is a target molecule for differentiation therapy of CIN.^11^ However, the factors that regulate FOXP4 expression in W12 cells remain unclear.

Previously, it was reported that the androgen/AR complex can regulate mRNA and protein expressions of Foxp1 and Foxp2 in the rat brain, proposing that androgen is an upstream regulator of Foxp.^14^ On the other hand, AR was reported to be expressed on the CIN lesions and AR expression becomes lost during CIN progression toward invasive SCC.^15^ Thus, androgens are presumed to be regulators of CIN differentiation at the early stages, leading to the strategy that intravaginal administration of androgens may be a nonsurgical differentiation therapy for CIN.

To test the above strategy, this study examined the effects of androgen on cell proliferation and squamous differentiation using AR‐transfected W12 cells. We also observed the role of androgen in FOXP4 function using AR‐transfected W12 cells and HaCaT cells, a human adult male skin‐derived keratinocyte cell line,^16^ which were reported to exhibit Ca^2+^‐ and FOXP4‐dependent squamous differentiation.^11^, ^17^, ^18^

## 2 MATERIALS AND METHODS

### 2.1 Cell lines

We obtained the human cervical dysplasia cell line W12 (RRID:CVCL_T290, clone 20,863), which contains HPV16 episomes, from Drs. Paul Lambert, Tomomi Nakahara, and Iwao Kukimoto.^19^ This cell line was authenticated by STR profiling and was cultured as previously reported.^11^

We obtained HaCaT (RRID:CVCL_0038), which is a spontaneously transformed immortal keratinocyte cell line derived from human adult male skin,^16^ from Cosmo Bio (Tokyo, Japan) and cultured it as previously reported.^11^

The human AR‐positive prostate cancer cell line LNCaP (RRID:CVCL_ 0395), which was authenticated by STR profiling, was gifted by Prof. Atsushi Mizokami, Department of Urology, Graduate School of Medical Sciences, Kanazawa University and was used for Western blot or RT‐PCR analysis as positive controls.

Mycoplasma infections were detected regularly and all experiments were performed under mycoplasma‐free conditions.

### 2.2 Samples

We obtained the uterine cervical tissues, normal (*n* = 5), CIN1 (*n* = 13), CIN2 (*n* = 14), CIN3 (*n* = 17), and SCC (*n* = 7), from 56 patients who had undergone hysterectomy or conization at Kanazawa University Hospital. These specimens were fixed with 20% formalin, embedded in paraffin, diagnosed, and used for the immunohistochemical study as previously reported.^11^ Clinical information of each sample is shown in Table 1.

**TABLE 1:** Clinical information of the patients included in this study.

Cervical tissue samples	Age	Pap test^a^	Preoperative diagnosis	Operative method	Postoperative diagnosis of cervical lesions
Normal‐#1 Normal‐#2 Normal‐#3 Normal‐#4 Normal‐#5	45 49 49 47 39	NILM NILM NILM NILM NILM	Leiomyoma Leiomyoma Adenomyosis Adenomyosis Adenomyosis	Hysterectomy Hysterectomy Hysterectomy Hysterectomy Hysterectomy	— — — — —
CIN1‐#1 CIN1‐#2 CIN1‐#3 CIN1‐#4 CIN1‐#5 CIN1‐#6 CIN1‐#7 CIN1‐#8 CIN1‐#9 CIN1‐#10 CIN1‐#11 CIN1‐#12 CIN1‐#13	41 64 39 40 48 48 35 40 34 33 48 30 59	LSIL AGC HSIL ASC‐US AGC/CIN1 HSIL HSIL HSIL HSIL LSIL HSIL ASC‐H LSIL	CIN2 AGC CIN2 CIN2 AGC/CIN1 CIN2‐3 CIN3 CIN3 CIN3 CIN2‐3 CIN2 CIN2 CIN3	Conization Conization Conization Conization Conization Conization Conization Conization Conization Conization Conization Conization Conization	CIN1 CIN1 CIN1 CIN1 CIN1 CIN1 CIN1/CIN3 CIN1/CIN3 CIN1/CIN3 CIN1 CIN1 CIN1 CIN1
CIN2‐#1 CIN2‐#2 CIN2‐#3 CIN2‐#4 CIN2‐#5 CIN2‐#6 CIN2‐#7 CIN2‐#8 CIN2‐#9 CIN2‐#10 CIN2‐#11 CIN2‐#12 CIN2‐#13 CIN2‐#14	40 35 42 46 28 42 40 40 49 55 32 27 38 31	HSIL HSIL ASC‐US HSIL AGC LSIL HSIL HSIL HSIL ASC‐H ASC‐US ASC‐H LSIL ASC‐US	CIN3 CIN3 CIN3 CIN3 AIS/CIN1 CIN2‐3 CIN3 CIN3 CIN2 CIN2 CIN2‐3 CIN2‐3 CIN3 CIN2	Conization Conization Conization Conization Conization Conization Conization Conization Conization Conization Conization Conization Conization Conization	CIN2/CIN3 CIN2/CIN3 CIN2/CIN3 CIN2 AIS/CIN2 CIN2/CIN3 CIN2 CIN2 CIN2 CIN2 CIN2 CIN2 CIN2 CIN2
CIN3‐#1 CIN3‐#2 CIN3‐#3 CIN3‐#4 CIN3‐#5 CIN3‐#6 CIN3‐#7 CIN3‐#8 CIN3‐#9 CIN3‐#10 CIN3‐#11 CIN3‐#12 CIN3‐#13 CIN3‐#14 CIN3‐#15 CIN3‐#16 CIN3‐#17	32 36 30 35 41 31 34 46 29 38 43 35 48 47 49 63 26	HSIL HSIL SCC HSIL HSIL HSIL HSIL HSIL HSIL HSIL HSIL SCC HSIL HSIL HSIL HSIL‐SCC HSIL	CIN3 CIN3 CIN3 CIN3 CIN2 CIN3 CIN3 CIN2 CIN3 CIN2 CIN3 CIN3 CIN3 CIN3 CIN3‐MIC CIN3‐MIC CIN2‐3	Conization Conization Conization Conization Conization Conization Conization Conization Conization Conization Conization Conization Conization Conization Conization Conization Conization	CIN3 CIN3 CIN3 CIN3 CIN3 CIN3 CIN3 CIN3 CIN3 CIN3 CIN3 CIN3 CIN3 CIN3 CIN3 CIN3 CIN3
SCC‐#1	48	SCC	IC	Radical hysterectomy	SCC (FIGO IIB^b^)
SCC‐#2	51	SCC	IC	Radical hysterectomy	SCC (FIGO IIIC1^b^)
SCC‐#3	58	HSIL	CIN3‐MIC	Conization	SCC (FIGO IA1^b^)
SCC‐#4	67	SCC	IC	Radical hysterectomy	SCC (FIGO IIIC1^b^)
SCC‐#5	47	SCC	IC	Radical hysterectomy	SCC(FIGO IIIC1^b^)
SCC‐#6	50	SCC	IC	Radical hysterectomy	SCC(FIGO IB3^b^)
SCC‐#7	49	SCC	IC	Radical hysterectomy	SCC(FIGO IIIC1^b^)

Abbreviations; IC, invasive cancer; MIC, microinvasive cancer.

^a^ Bethesda System 2001.

^b^ FIGO 2018.

### 2.3 Immunohistochemical and immunocytochemical staining

Immunohistochemical staining was performed using the avidin–biotin–peroxidase complex method according to the manufacturer's instructions (VECTASTAIN ABC Kit, Vector Laboratories, ) as described previously.^11^ The slides were incubated overnight at 4°C with either one of primary antibodies, anti‐AR rabbit monoclonal antibody (1:200, clone SP107, RRID: AB_2537931, ab105225, Abcam) or anti‐p63 mouse monoclonal antibody (1:100 clone 4A4, RRID: AB_305870, ab735, Abcam). Control staining was performed by replacing the primary antibody with a normal rabbit or mouse serum solution.

The W12 cells or HaCaT cells transfected with pCAGIPuro or pCAGIPuro‐AR were cultured on the glass chamber slide (Lab‐Tek®II, Thermo Fisher Scientific Inc.). The slides with 80% confluent cells were fixed in 4% paraformaldehyde and were incubated overnight at 4°C with anti‐FOXP4 rabbit polyclonal antibody (1:200, HPA007176, RRID: AB_1078911, Sigma‐Aldrich) or anti‐AR mouse monoclonal antibody (1:100, clone AR441, RRID: AB_11000751, Dako, Tokyo, Japan). Control staining was performed by normal rabbit or mouse serum solution. Secondary labelling was with Alexa Fluor™ 488‐labeled goat anti‐mouse IgG antibody (A‐11001, Invitrogen.) or Alexa Fluor™ 555‐labeled goat anti‐rabbit IgG antibody (A32732, Invitrogen), respectively.

### 2.4 Assessment of AR expression profiles in CIN lesions

The AR‐positive rates in CIN lesions were analyzed and values were normalized using NIH ImageJ software as described previously.^11^, ^20^

### 2.5 Western blot analysis

Western blot analysis was performed as described previously.^11^ Protein lysates were extracted with RIPA buffer (Cell Signaling Technology Inc.), and were electrophoresed on a 7.5% SDS‐PAGE gel and then transferred to a nitrocellulose membrane. The transferred membranes were incubated with primary antibody against FOXP4 (1:1000, RRID: AB_2262825, 16,772‐1‐AP, Proteintech Group Inc. ), AR (1:5000, the rabbit polyclonal antibody NH27, kindly provided by Prof. Atsushi Mizokami of Kanazawa University), or β‐Actin (1:5000, RRID: AB_630835, C‐11, Santa Cruz Biotechnology) overnight at 4°C. A secondary horse‐radish peroxidase conjugated antibody was applied for 1 h at room temperature. The blots were visualized as described previously.^11^

### 2.6 RT‐PCR and quantitative real‐time PCR analysis

RT‐qPCR was performed as described previously.^11^ The cDNA was amplified using specific primers, forward (Fd) and reverse (Rs) primers (Table 2), and hypoxanthine phosphoribosyltransferase 1 (HPRT1) was used for the control. The results were shown as mean ± SD of three independent experiments.

**TABLE 2:** Primer sequences for RT‐PCR and quantitative real‐time PCR.

Gene	Forward primers	Reverse primers
AR	AGGATGCTCTACTTCGCCCC	CTGGCTGTACATCCGGGAC
Foxp4	GTTCACCAGGATGTTCGCCT	CTCCGCTTCTGATACTCCCG
KRT1	ATTTCTGAGCTGAATCGTGTGATC	CTTGGCATCCTTGAGGGCATT
KRT10	TGATGTGAATGTGGAAATGAATGC	GTAGTCAGTTCCTTGCTCTTTTCA
IVL	GGGTGGTTATTTATGTTTGGGTGG	GCCAGGTCCAAGACATTCAAC
Hes1	AGCTGGAGAAGGCGGACATT	CATTGATCTGGGTCATGCAG
Hes2	AGAACTCCAACTGCTCGAAGC	CGGTCATTTCCAGGACGTCT
Hes5	CCGGTGGTGGAGAAGATG	TAGTCCTGGTGCAGGCTCTT
Hey1	GAGAAGCGCCGACGAGACCG	GGCGTGCGCGTCAAAGTAACCTTT
ELF3	CAACTATGGGGCCAAAAGAA	TTCCGACTCTGGAGAACCTC
TGM1	TCAGACGCTGGGGAGTTC	GGTCCGCTCACCAATCTG
GRHL3	GCCAGTTCTACCCCGTCA	GTCAATGACCCGCTGCTT
SPRR1	CCAGCAGAAGACCAAGCAGAA	GCAAATGGGACTCATACGCAGAATG
NOTCH1	CAATGTGGATGCCGCAGTTGTG	CAGCACCTTGGCGGTCTCGTA
NOTCH2	AAAAATGGGGCCAACCGAGAC	TTCATCCAGAAGGCGCACAA
NOTCH3	AGATTCTCATCCGAAACCGCTCTA	GGGGTCTCCTCCTTGCTATCCTG
NOTCH4	CAGCCCAAGCAGATATGTAAGGA	CGTCCAACCCACGTCACA
HPRT1	GCCCTGGCGTCGTGATTAGT	CGAGCAAGACGTTCAGTCCTGTC

### 2.7 AR transfection

The empty vector (pCAGIPuro) was created by excluding the flag‐Myc fragment from pCAGIPuro‐FlagmcMycWT with *Xho*I cutting and self‐ligation (Clone ID; RDB_14100, RIKEN DNA BANK).^21^ To create an AR expression vector (pCAGIPuro‐AR), a 1.4‐kbp human AR fragment prepared from pEGFP‐AR (kindly provided by Prof. Atsushi Mizokami of Kanazawa University) with *Bgl*II and *Bam*HI was subcloned into pCAGIPuro. W12 cells or HaCaT cells were seeded in 6‐well plates at 5 × 10^5^ cells per well. After culture overnight, the cells were transfected with pCAGIPuro or pCAGIPuro‐AR using GenomeOne‐GX (Ishihara Sangyo Kaisha, Ltd.) and KALA amphipathic peptide (Cosmo Bio Co., LTD) according to the manufacturer's protocol. After 48 h, 1 μg/μL puromycin (Thermo Fisher Scientific) was added to the cells for 7 days to establish stable cell lines.

### 2.8 Lentiviral transfection

RNA interference for reduction of FOXP4 gene expression was performed using lentivirus particles of MISSION®small hairpin RNA (FOXP4‐shRNA1‐5: TRCN0000285257, TRCN0000274833, TRCN0000274834, TRCN0000274832, and TRCN0000274894, Sigma‐Aldrich) as described previously.^11^

### 2.9 siRNA transfection

Transfection of siRNA into the W12 cells was performed as described previously.^11^ siRNAs for *FOXP4* (s41928 and s41929, Thermo Fisher Scientific), *ELF3* (s4623 and s4624, Thermo Fisher Scientific), *GRHL3* (s33752 and s33754, Thermo Fisher Scientific), *NOTCH3* (s9640 and s9641, Thermo Fisher Scientific), and corresponding Silencer® Select Negative Control No.1 (AM4635, Thermo Fisher Scientific) were used.

### 2.10 Cell proliferation assay

Cell proliferation assay was performed in triplicate using Cell Proliferation Reagent WST‐1 (Roche, Mannheim, Germany) as described previously.^11^

### 2.11 Immunocytochemical assay of extranuclear distribution of FOXP4

After AR‐transfected W12 cells were treated with DHT (10 nM), FOXP4 was immunocytologically stained. The FOXP4‐positive extranuclear areas were detected by subtracting Hoechst‐positive nuclear regions and were calculated by NIH ImageJ software.

### 2.12 Statistical analysis

In order to assess differences in the AR‐positive area ratio among CIN stages, Kruskal–Wallis followed by Steel‐Dwass was performed. The data are shown as the median and interquartile range. The differences in FOXP4‐positive extranuclear areas between DHT‐stimulated and nonstimulated W12 cells, and the differences in the expression of FOXP4, squamous differentiation‐related genes between the treated and non‐treated cells, and cell proliferation assay were evaluated by the paired *t‐*test or ANOVA followed by the Dunnett post hoc test (SPSS Statistics version 25.0, IBM, USA). *p*‐values of <0.05 were considered significant.

## 3 RESULTS

### 3.1 AR transfection in W12 cells

**FIGURE 1:**
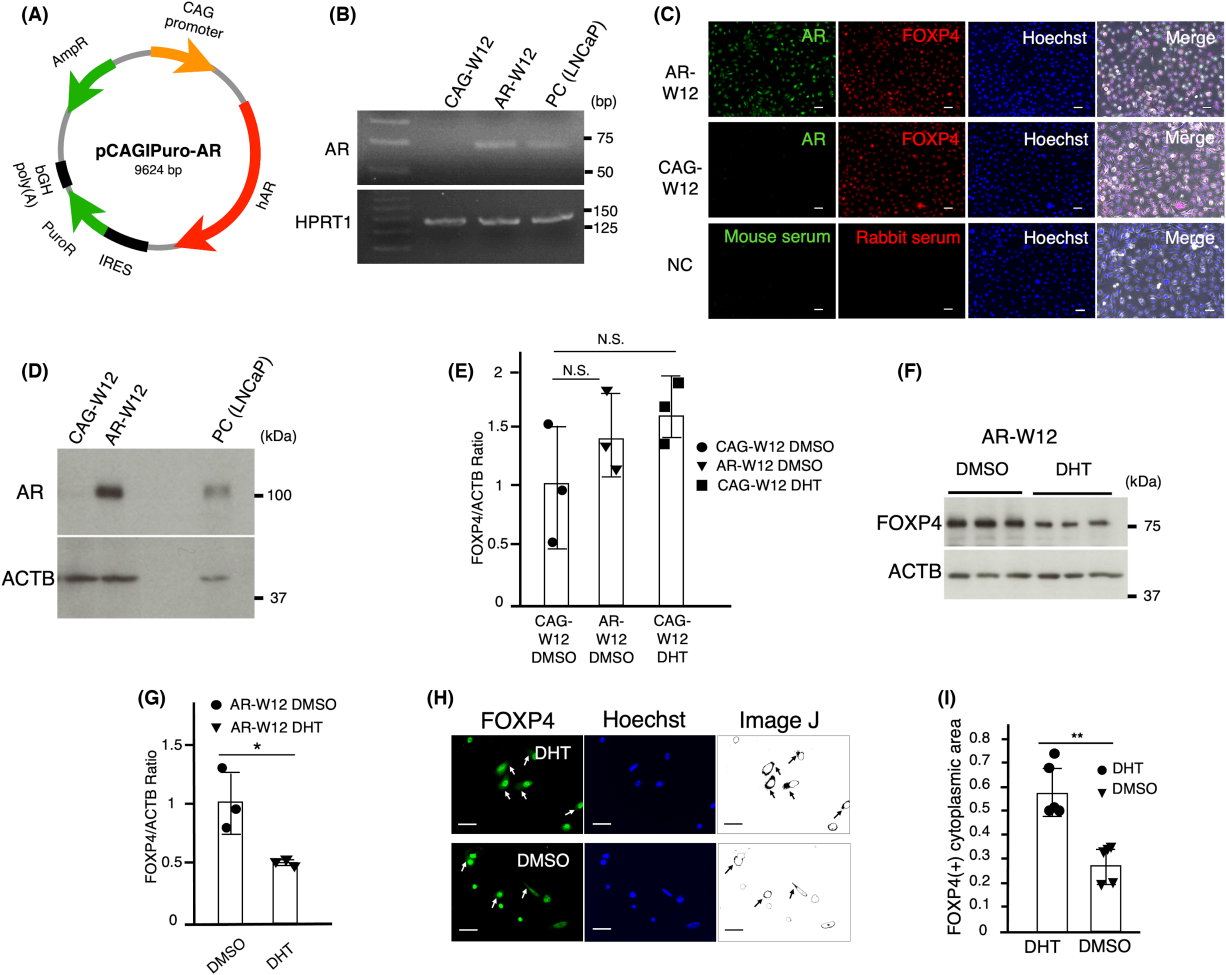
DHT affects FOXP4 expression in AR‐transfected W12 cells. (A) Since AR expression was hardly detected in W12 cells, these cells were transfected with AR using a CAG promoter. (B) RT‐PCR confirmed the mRNA expression of AR in AR‐transfected W12 cells. (C) Immunocytochemical staining showed the intranuclear localization of AR and FOXP4 in AR‐transfected W12 cells. Scale bars show 50 μm. (D) Western blot analysis confirmed protein expression of transfected AR. LNCaP, an AR‐positive prostate cancer cell line, was used for positive control. (E) Western blot analysis showed that FOXP4 expression was not significantly changed by the transfection of the AR gene. DHT (10 nM) did not significantly change FOXP4 expression in CAG‐transfected W12 cells. (F, G) DHT treatment significantly reduced protein expression of FOXP4 in AR‐transfected W12 cells. (H) Extranuclear distribution of FOXP4 in W12 cells evaluated by ImageJ. Scale bars show 50 μm. (I) DHT (10 nM) treatment significantly promoted the FOXP4‐positive cytoplasmic area in AR‐transfected W12 cells. Five separate experiments were performed and bars represent SD. The data were analyzed by paired *t*‐test and ANOVA followed by the Dunnett post hoc test. *p < 0.05; **p < 0.01; N.S., not significant.

Since AR expression was hardly detected in W12 cells, these cells were transfected with AR using a CAG promoter (Figure 1A). RT‐PCR confirmed the mRNA expression of AR in AR‐transfected W12 cells (Figure 1B). Immunocytochemical staining showed the intranuclear localization of AR and FOXP4 in AR‐transfected W12 cells (Figure 1C). Protein expression of transfected AR was confirmed by Western blot analysis (Figure 1D). LNCaP, an AR‐positive prostate cancer cell line, was used for positive control.

### 3.2 DHT impairs FOXP4 expression in AR‐transfected W12 cells

Western blot analysis showed that FOXP4 expression was not significantly changed by transfection of the AR gene (Figure 1E). The treatment of DHT (10 nM) did not significantly change the protein expression of FOXP4 in CAG‐transfected W12 cells (Figure 1E), whereas it reduced FOXP4 expression in AR‐transfected W12 cells (Figure 1F,G). This treatment promoted the extranuclear distribution of FOXP4, suggesting the inhibitory effects of androgen on FOXP4 functions (Figure 1H,I).

### 3.3 DHT inhibits proliferation and promotes squamous differentiation in AR‐transfected W12 cells

**FIGURE 2:**
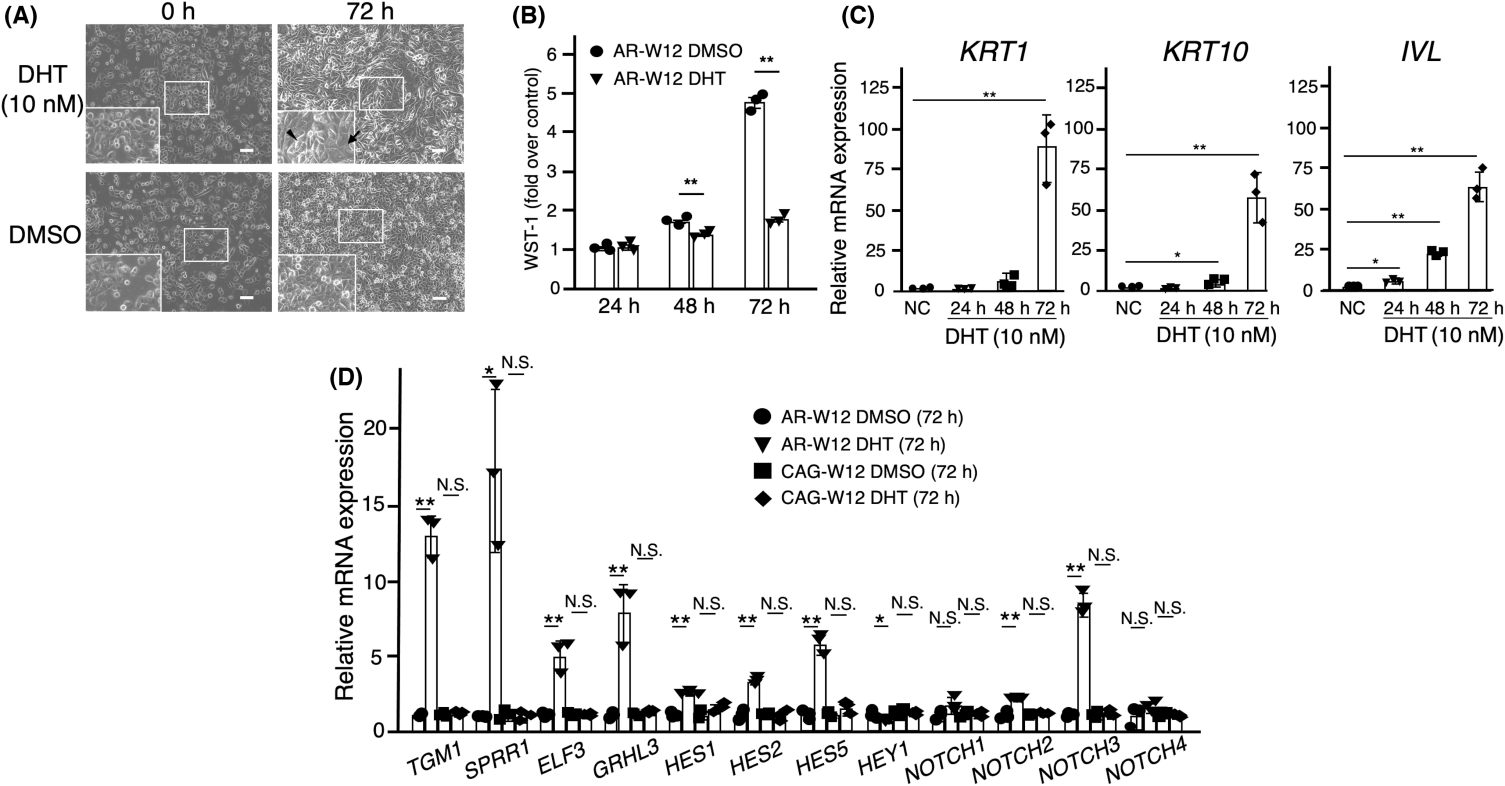
DHT inhibits proliferation and promotes squamous differentiation in AR‐transfected W12 cells. (A) DHT‐treatment induced mature squamous phenotypes in W12 cells, showing a flattened appearance (arrow) and stratified growth (arrowhead). Scale bars show 100 μm. (B) DHT decreased the proliferation of W12 cells during 72‐h incubation. The results are shown as means of individual experiments (*N* = 3) and bars represent SEM. The data were analyzed by paired *t*‐test. (C) DHT increased mRNA expressions of *KRT1*, *KRT10*, and *IVL*. (D) DHT increased mRNA expressions of squamous differentiation‐related genes, *TGM1*, *SPRR1*, *ELF3*, *GRHL3*, *HES1*, *HES2*, *HES5*, *NOTCH2*, and *NOTCH3*, but not *NOTCH1* nor *NOTCH4* in AR‐transfected W12 cells. Three separate experiments were performed and bars represent SD. The data were analyzed by ANOVA followed by the Dunnett post hoc test. *p < 0.05; **p < 0.01; N.S., not significant.

DHT treatment induced mature squamous phenotypes, showing a flattened appearance (arrow) and stratified growth (arrowhead) in places (Figure 2A). This treatment significantly decreased the proliferation of W12 cells during 72‐h incubation (Figure 2B). RT‐qPCR analysis demonstrated significant increases in mRNA expressions of squamous differentiation markers, *KRT1*, *KRT10*, and *IVL*, in AR‐transfected W12 cells (Figure 2C).

In a previous study, we reported that the downregulation of FOXP4 induced squamous differentiation‐ and NOTCH signal‐related gene expressions.^11^ In this study, DHT administration significantly promoted mRNA expression of *TGM1*, *SPRR1*, *ELF3*, *GRHL3*, *HES1*, *HES2*, *HES5*, *NOTCH2*, and *NOTCH3*, but not *NOTCH1* nor *NOTCH4* (Figure 2D).

### 3.4 Downregulation of *ELF3* inhibits androgen‐promoted squamous differentiation

**FIGURE 3:**
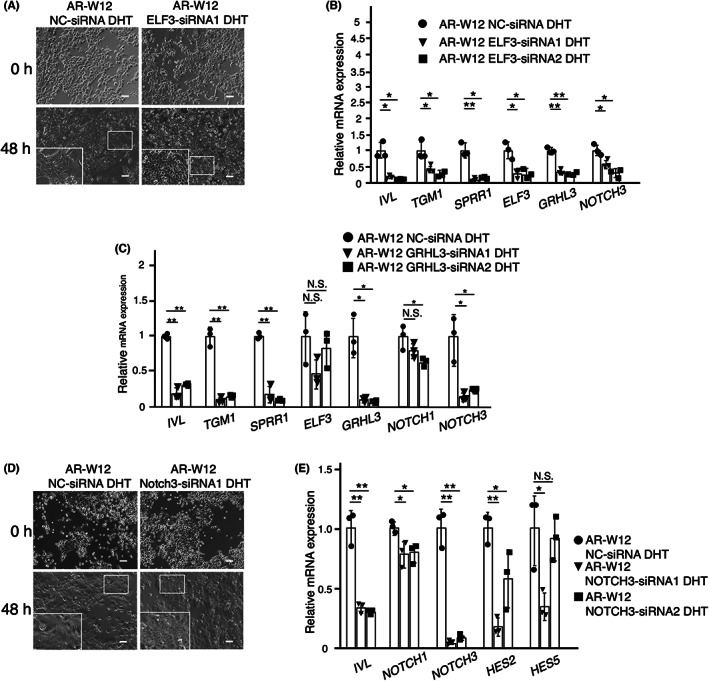
*ELF3*, *GRHL3*, and *NOTCH3* are involved in androgen‐promoted squamous differentiation. (A) Knockdown of *ELF3* by siRNAs inhibited morphological changes in squamous differentiation induced by DHT. Scale bars show 100 μm. (B) Knockdown of *ELF3* also attenuated DHT‐induced gene expressions of the transcriptional factor *GRHL3* and squamous differentiation markers *TGM1*, *SPRR1*, and *IVL* as well as *NOTCH3* in DHT‐treated W12 cells. (C) Knockdown of *GRHL3* inhibited gene induction of *TGM1*, *SPRR1*, *NOTCH3*, and *IVL* in DHT‐treated W12 cells. (D) Knockdown of *NOTCH3* in DHT‐treated W12 cells inhibited morphological changes in squamous differentiation. Scale bars show 100 μm. (E) Knockdown of *NOTCH3* attenuated DHT‐induced gene expression of *HES2* and *IVL*. Three separate experiments were performed and the bars represent SD. The data were analyzed by ANOVA followed by the Dunnett post hoc test. *p < 0.05; **p < 0.01; N.S., not significant.

Knockdown of *ELF3* by siRNAs inhibited morphological changes in squamous differentiation in DHT‐treated W12 cells (Figure 3A). This treatment also inhibited the expression of differentiation‐related genes induced by DHT (Figure 3B).

### 3.5 Downregulation of *GRHL3* or *NOTCH3* inhibits androgen‐promoted squamous differentiation

Knockdown of *GRHL3* inhibited gene induction of *TGM1*, *SPRR1*, *NOTCH3*, and *IVL* in DHT‐treated W12 cells (Figure 3C). Additional application of siRNAs targeting *NOTCH3* showed inhibition of morphological changes in squamous differentiation induced by DHT (Figure 3D) and attenuated gene induction of *HES2* and *IVL* (Figure 3E). These results suggest that *GRHL3* and *NOTCH3* are key molecules for DHT‐induced squamous differentiation.

### 3.6 DHT inhibits FOXP4 expression in AR‐transfected HaCaT cells

**FIGURE 4:**
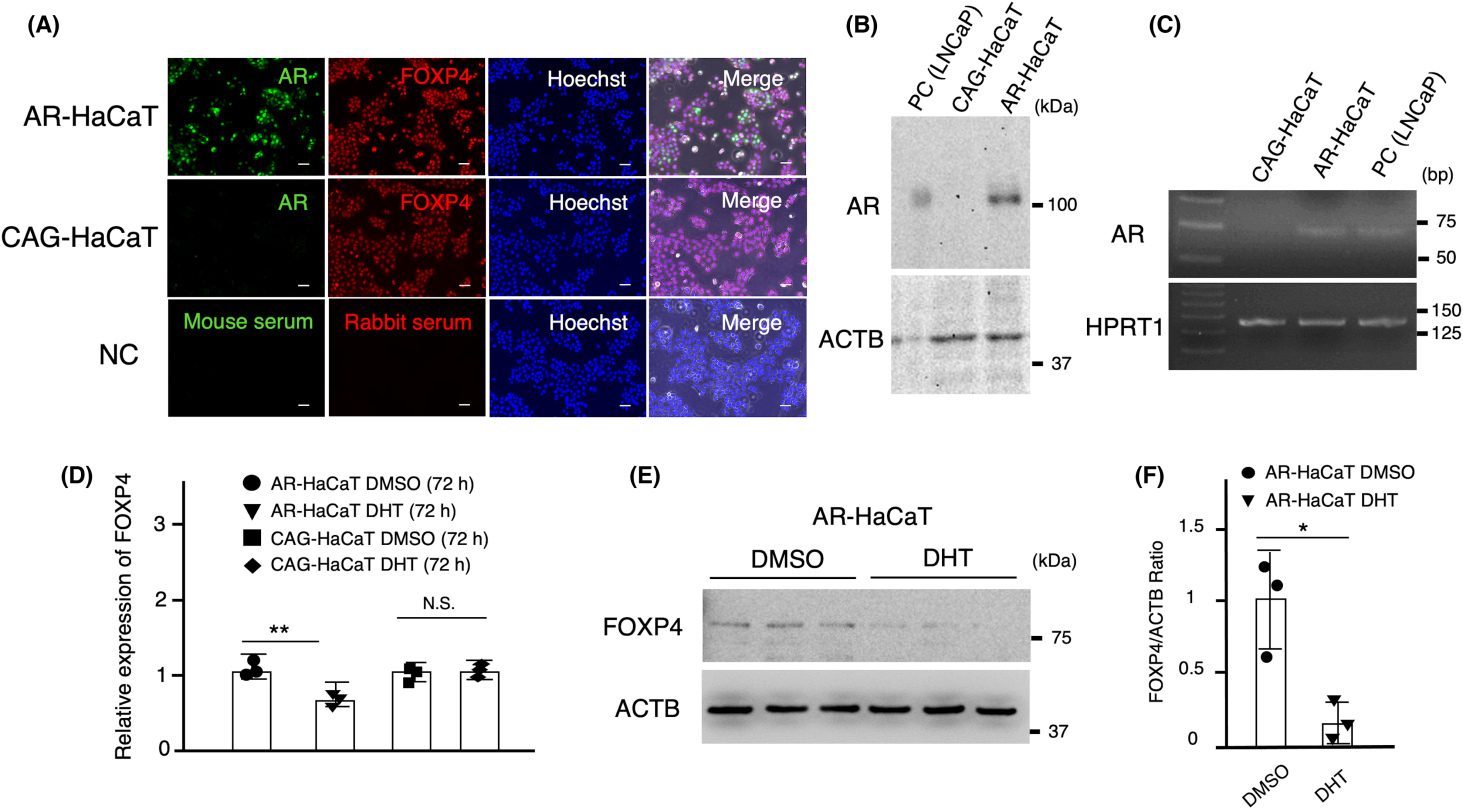
DHT inhibits *FOXP4* expression in AR‐transfected HaCaT cells. (A) In the recommended maintaining culture condition (1.8 mM CaCl_2_), intranuclear localization of AR in AR‐transfected HaCaT cells was verified by immunofluorescence staining. Scale bars show 50 μm. (B) Western blot analysis showed protein expression of transfected AR. (C) RT‐PCR confirmed the mRNA expression of AR in AR‐transfected W12 cells. (D) DHT (10 nM) reduced the mRNA expression of FOXP4 during a 72‐h culture. (E, F) Western blot analysis showed that DHT (10 nM) significantly decreased the protein expression of FOXP4. Three separate experiments were performed and bars represent SD. The data were analyzed by paired *t*‐test. *p < 0.05; **p < 0.01; N.S., not significant.

To examine whether ARs are generally involved in squamous differentiation, we investigated the effects of DHT on FOXP4 expression in the HaCaT cell line, one of the most used cell lines in the area of squamous differentiation research. Under the recommended Ca^2+^ concentration (1.8 mM CaCl_2_), intranuclear localization of AR in AR‐transfected HaCaT cells was verified by immunofluorescence staining (Figure 4A). Protein expression of transfected AR was confirmed by Western blot analysis (Figure 4B). RT‐PCR confirmed the mRNA expression of AR in AR‐transfected W12 cells (Figure 4C). DHT (10 nM) reduced mRNA and protein expressions of FOXP4 during 72‐h culture (Figure 4D–F).

### 3.7 AR is expressed in atypical cells in CIN lesions

**FIGURE 5:**
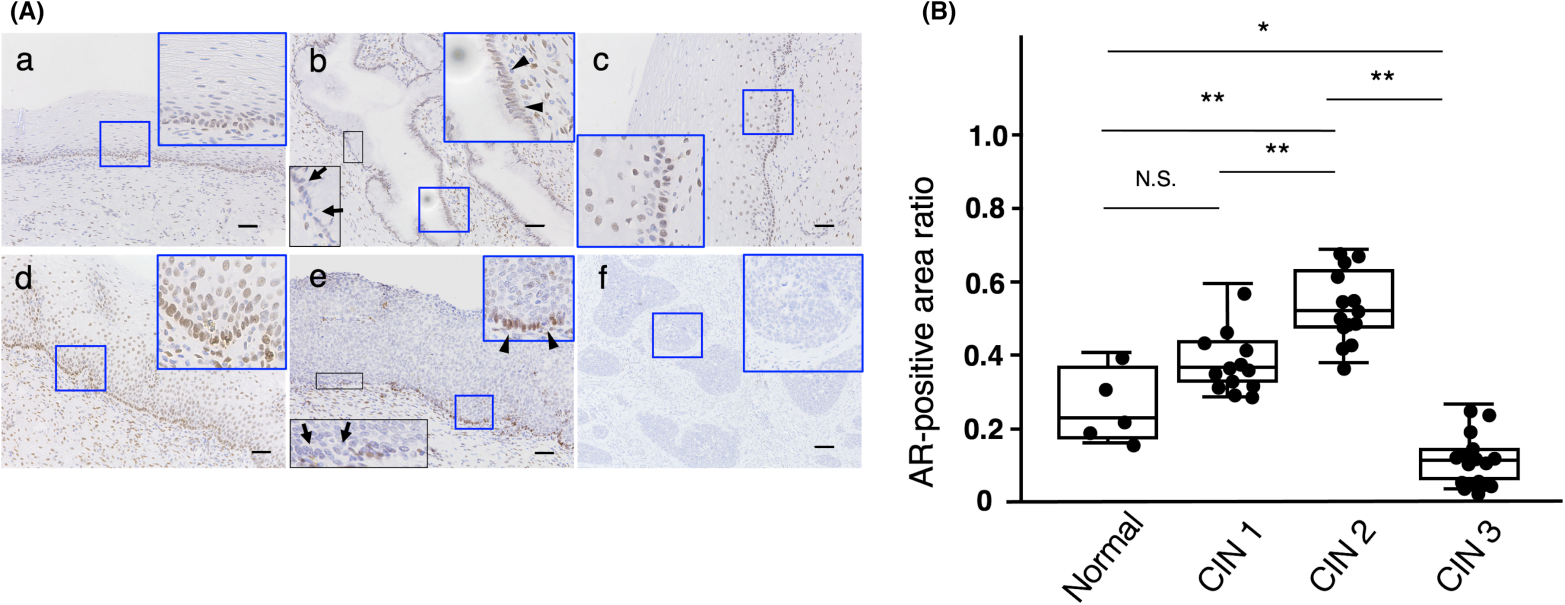
The expression profiles of AR on normal tissues and CIN and SCC lesions by immunohistochemistry. (A) AR was expressed in the basal to parabasal layers of the normal cervical epithelium (a). In endocervical epithelial cells, AR expression was observed (arrowheads), but partially negative (arrows) (b). In CIN1 (c) and CIN2 (d) lesions, AR was detected in the atypical squamous cells. (e) AR expression had almost disappeared in the CIN3 lesion except for the basal layer (arrowheads), whose expression was partially abolished (arrows). (f) AR expression was not detected in SCC. Scale bars show 50 μm. (B) AR‐positive areas significantly increased from normal to CIN2 and decreased from CIN2 to CIN3. The data were analyzed by Kruskal–Wallis followed by Steel‐Dwass and shown as the median and interquartile range. *p < 0.05; **p < 0.01; N.S., not significant.

An immunohistochemical study showed that AR was expressed in the basal to parabasal layers of the normal cervical epithelium (Figure 5A‐a). In endocervical epithelial cells, AR expression was observed (arrowheads), but partially negative (arrows) (Figure 5A‐b). In CIN1 and 2 lesions, AR was detected in the atypical squamous cells (Figure 5A‐c,d), whereas AR expression had almost disappeared in the CIN3 lesion except for the basal layer (Figure 5A‐e, arrowheads), whose expression was partially abolished (Figure 5A‐e, arrows). AR expression was not detected in any cases of SCC (Figure 5A‐f).

AR‐positive image analysis confirmed that AR‐positive areas significantly increased from normal to CIN2 and decreased from CIN2 to CIN3 (Figure 5B).

## 4 DISCUSSION

It is well known that estrogens stimulate the proliferation of stratified squamous epithelium of the ectocervix.^22^ In contrast, this study demonstrated that DHT attenuated cell proliferation using AR‐transfected W12 cells, suggesting that androgen acts antagonistically to estrogen in the stratified squamous cells of the cervix. We also showed that DHT promoted morphological changes that mimic squamous differentiation, and significantly increased the gene expressions of squamous differentiation markers, *KRT1*, *KRT10*, and *IVL*, suggesting that DHT can inhibit the proliferation and promote the differentiation of atypical squamous cells in CIN lesions. To support the different effects of sex steroid hormones on CIN, it was reported that prolonged exposure to estrogen induces carcinogenesis within the cervical and vaginal squamous epithelium using transgenic mice expressing the oncogenes of HPV16.^23^ Estrogen receptor (ER) antagonists and selective ER modulators were also demonstrated to be candidates to protect cancer progression in both the cervix and the vagina.^24^

By immunohistochemical examination, this study showed that AR was expressed in the basal to parabasal layers of the normal cervical epithelium. In CIN1 and CIN2 lesions, AR was detected in the atypical squamous cells, whereas AR expression had almost disappeared in the CIN3 lesion and was not detected in SCC. These expression profiles are compatible with previous reports^15^ and suggest that the androgens do not act to promote squamous differentiation in the late stages of CIN.

We recently reported that FOXP4 was expressed in atypical squamous cells in CIN and that downregulation of FOXP4 attenuated the proliferation of W12 cells, proposing that FOXP4 is a target molecule for differentiation therapy of CIN.^11^ This study revealed that DHT attenuated the protein expression of FOXP4 and reduced its intranuclear distribution in AR‐transfected W12 cells. It was also demonstrated that DHT reduced the expression of FOXP4 in AR‐transfected HaCaT cells, supporting that androgen can regulate FOXP4 function. These findings also suggest that ARs are generally involved in squamous differentiation.

Transcription factor ELF3 was reported to induce expression of NOTCH3^25^ and the HES family.^26^ Somatic mutation or reduced expression of ELF3 was reported in uterine cervical adenocarcinomas,^27^ urothelial bladder carcinoma,^28^ and oral SCC.^29^ We previously showed that the downregulation of FOXP4 induced squamous differentiation in W12 cells via an ELF3‐dependent pathway. Consistent with it, this study showed that DHT induced the expression of squamous differentiation‐related genes in AR‐transfected W12 cells via an ELF3‐dependent pathway, indicating that ELF3 is a common molecular target for FOXP4‐ and androgen‐dependent differentiation therapy of CIN. Our preliminary study using microarray analyses showed several molecules were commonly downregulated by both knockdown of FOXP4 and administration of DHT (unpublished data). Consequently, these in vitro models may be useful to detect new candidates for target molecules to develop differentiation therapies of CIN.

This study supports the previous concept that the sex hormone environment is involved in the progression of CIN.^30^, ^31^ In women, a high level of endogenous estradiol was reported to be a risk for HPV16‐induced onset of uterine cervix squamous carcinoma,^30^ whereas the other study showed no significant relationship between CIN and endogenous sex steroid hormones such as estradiol, estrone, estrone‐sulfate, dehydroepiandrosterone sulfate, and progesterone.^32^ However, there is little information about the pathophysiological effects of androgen on CIN progression. In this regard, this study provides a new viewpoint that androgen is another important hormonal factor for CIN differentiation.

Since differentiation therapy by all‐*trans* retinoic acid is effective for patients with acute promyelocytic leukemia,^33^, ^34^ several clinical approaches of differentiation therapy for CIN were tried using retinoids and showed some effect to block CIN2 progression.^35^, ^36^ However, they had no effects on CIN3 progression.^35^, ^37^ Two decades ago, based on the immune‐modulatory and tumor‐inhibitory activity of dehydroepiandrosterone, an adrenal androgen, against skin papillomas and carcinoma,^38^, ^39^ a pilot study was conducted for 12 women with low‐grade CIN and CIN regression was observed in 10 of the 12 women after intravaginal administration of dehydroepiandrosterone for 6 months.^40^ Although it is unclear whether this regression rate is significant or not as compared with the spontaneous regression rate, this study reported that long‐term intravaginal administration of dehydroepiandrosterone had no side effects. Taken together, considering that AR is constantly expressed on the basal layer of the stratified squamous cells and CIN1 and CIN2 lesions, local intravaginal administration of androgen is a promising approach to the early stage of CIN from the new perspective of differentiation therapy.

A major limitation of our study is the lack of animal experiments. Since animal cells are not natural hosts for HPV, the use of an animal model of CIN under HPV infection is difficult. Androgen effects should be investigated using transgenic mice expressing the oncogenes of HPV16 in the future.^23^ Second, we should take into account the possibility that the forced expression of AR can cause artifacts in cell functions.

**FIGURE 6:**
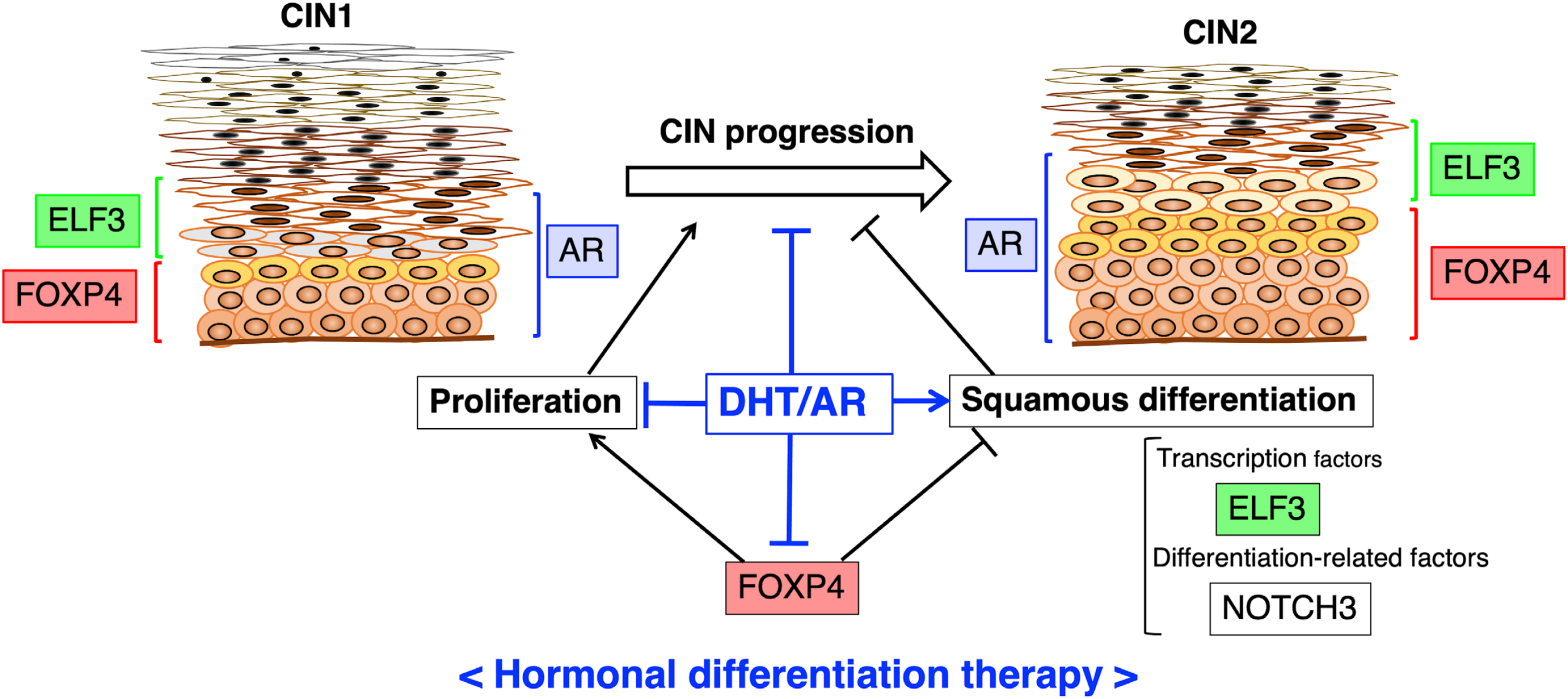
Estimated roles of androgen/AR in FOXP4‐related proliferation and squamous differentiation in the uterine cervix. We previously proposed that FOXP4 promotes proliferation and inhibits squamous differentiation through ELF3 and NOTCH3 in human uterine cervical epithelial cells, indicating that FOXP4 is a candidate target molecule for differentiation therapy of CIN. This study showed that DHT/AR promotes squamous differentiation and inhibits proliferation in W12 cells partially through regulating the FOXP4 function (shown in blue), suggesting the significant role of hormonal environment in CIN progression. These findings suggest that DHT/AR are new candidate molecules for differentiation therapy of CIN.

In conclusion, as we summarize the novel findings in Figure 6, this study showed that DHT attenuated proliferation and promoted the expression of squamous differentiation‐related genes in AR‐transfected W12 cells. It also indicated that ELF3 is a common molecular target for FOXP4‐ and androgen‐dependent differentiation therapy of CIN. This study supports that the sex hormone environment is involved in the progression of CIN and throws a new viewpoint that androgen is another important hormonal factor for CIN regulation. Based on these results, we propose that androgen is a novel factor that regulates CIN differentiation, providing a new strategy for nonsurgical and hormone‐induced differentiation therapy against CIN1 and CIN2.

## AUTHOR CONTRIBUTIONS

**Takeo Matsumoto:** Data curation (equal); formal analysis (equal); investigation (equal); resources (equal); writing – original draft (equal). **Takuma Suzuki:** Data curation (equal); formal analysis (equal); investigation (equal). **Mitsuhiro Nakamura:** Conceptualization (equal); resources (equal); supervision (equal). **Megumi Yamamoto:** Data curation (supporting); investigation (supporting). **TAKASHI IIZUKA:** Data curation (supporting); formal analysis (supporting); investigation (supporting). **Masanori Ono:** Resources (supporting). **Kyosuke Kagami:** Data curation (supporting); investigation (supporting). **Haruki Kasama:** Data curation (supporting); investigation (supporting). **Tatsuhito Kanda:** Data curation (supporting); investigation (supporting). **Yuya Sakai:** Data curation (supporting); investigation (supporting). **Jyunpei Iwadare:** Resources (supporting). **Ayumi Matsuoka:** Data curation (supporting); investigation (supporting). **Kayo Kayahashi:** Data curation (supporting); investigation (supporting). **Kousho Wakae:** Supervision (supporting). **Masamichi Muramatsu:** Supervision (supporting). **Satoru Kyo:** Supervision (supporting). **Yasuhiko Yamamoto:** Supervision (supporting). **Yasunari Mizumoto:** Conceptualization (equal); resources (equal); supervision (equal). **Takiko Daikoku:** Conceptualization (equal); funding acquisition (equal); supervision (equal).

## FUNDING INFORMATION

This work was supported in part by a Grants‐in‐Aid for Scientific Research (nos. 17H04337, 19H01617, 19K22681, 20H03822, and 21H04837) and the Japan Agency for Medical Research and Development (no. 20ck0106549h0001).

## CONFLICT OF INTEREST STATEMENT

The authors declare no competing interests.

## ETHICS APPROVAL AND CONSENT TO PARTICIPATE

All protocols were approved by the Medical Ethics Committee of Kanazawa University (approval number: 2015‐085). Human studies were conducted according to the Declaration of Helsinki principles, and written informed consent was received from participants prior to inclusion in the study.

## Data Availability

All data generated or analyzed during this study are included in this published article. The data that support the findings of this study are available from the corresponding author upon reasonable request.

## References

[1] Walboomers JM , Jacobs MV , Manos MM , et al. Human papillomavirus is a necessary cause of invasive cervical cancer worldwide. J Pathol. 1999;189(1):12‐19. 10451482 10.1002/(SICI)1096-9896(199909)189:1<12::AID-PATH431>3.0.CO;2-F

[2] Drolet M , Benard E , Perez N , Brisson M , HPV Vaccination Impact Study Group . Population‐level impact and herd effects following the introduction of human papillomavirus vaccination programmes: updated systematic review and meta‐analysis. Lancet. 2019;394(10197):497‐509. 31255301 10.1016/S0140-6736(19)30298-3 PMC7316527

[3] Garbuglia AR , Lapa D , Sias C , Capobianchi MR , Del Porto P . The use of both therapeutic and prophylactic vaccines in the therapy of papillomavirus disease. Front Immunol. 2020;11:188. 32133000 10.3389/fimmu.2020.00188 PMC7040023

[4] Gupta SM , Mania‐Pramanik J . Molecular mechanisms in progression of HPV‐associated cervical carcinogenesis. J Biomed Sci. 2019;26(1):28. 31014351 10.1186/s12929-019-0520-2 PMC6477741

[5] Buckley CH , Butler EB , Fox H . Cervical intraepithelial neoplasia. J Clin Pathol. 1982;35(1):1‐13. 7037858 10.1136/jcp.35.1.1 PMC497441

[6] Zehbe I , Richard C , DeCarlo CA , et al. Human papillomavirus 16 E6 variants differ in their dysregulation of human keratinocyte differentiation and apoptosis. Virology. 2009;383(1):69‐77. 18986660 10.1016/j.virol.2008.09.036 PMC2945146

[7] Nees M , Geoghegan JM , Munson P , et al. Human papillomavirus type 16 E6 and E7 proteins inhibit differentiation‐dependent expression of transforming growth factor‐beta2 in cervical keratinocytes. Cancer Res. 2000;60(15):4289‐4298. 10945644

[8] White EA . Manipulation of epithelial differentiation by HPV Oncoproteins. Viruses. 2019;11(4):369. 31013597 10.3390/v11040369 PMC6549445

[9] Hatterschide J , Bohidar AE , Grace M , et al. PTPN14 degradation by high‐risk human papillomavirus E7 limits keratinocyte differentiation and contributes to HPV‐mediated oncogenesis. Proc Natl Acad Sci U S A. 2019;116(14):7033‐7042. 30894485 10.1073/pnas.1819534116 PMC6452706

[10] Meyers JM , Uberoi A , Grace M , Lambert PF , Munger K . Cutaneous HPV8 and MmuPV1 E6 proteins target the NOTCH and TGF‐beta tumor suppressors to inhibit differentiation and sustain keratinocyte proliferation. PLoS Pathog. 2017;13(1):e1006171. 28107544 10.1371/journal.ppat.1006171 PMC5287491

[11] Matsumoto T , Iizuka T , Nakamura M , et al. FOXP4 inhibits squamous differentiation of atypical cells in cervical intraepithelial neoplasia via an ELF3‐dependent pathway. Cancer Sci. 2022;113:3376‐3389. 35838233 10.1111/cas.15489 PMC9530870

[12] Golson ML , Kaestner KH . Fox transcription factors: from development to disease. Development. 2016;143(24):4558‐4570. 27965437 10.1242/dev.112672 PMC5201025

[13] Hannenhalli S , Kaestner KH . The evolution of Fox genes and their role in development and disease. Nat Rev Genet. 2009;10(4):233‐240. 19274050 10.1038/nrg2523 PMC2733165

[14] Bowers JM , Perez‐Pouchoulen M , Roby CR , Ryan TE , McCarthy MM . Androgen modulation of Foxp1 and Foxp2 in the developing rat brain: impact on sex specific vocalization. Endocrinology. 2014;155(12):4881‐4894. 25247470 10.1210/en.2014-1486 PMC4239422

[15] Noel JC , Bucella D , Fayt I , et al. Androgen receptor expression in cervical intraepithelial neoplasia and invasive squamous cell carcinoma of the cervix. Int J Gynecol Pathol. 2008;27(3):437‐441. 18580324 10.1097/PGP.0b013e318160c599

[16] Boukamp P , Petrussevska RT , Breitkreutz D , Hornung J , Markham A , Fusenig NE . Normal keratinization in a spontaneously immortalized aneuploid human keratinocyte cell line. J Cell Biol. 1988;106(3):761‐771. 2450098 10.1083/jcb.106.3.761 PMC2115116

[17] Choo CK , Rorke EA , Eckert RL . Differentiation‐independent constitutive expression of the human papillomavirus type 16 E6 and E7 oncogenes in the CaSki cervical tumour cell line. J Gen Virol. 1994;75(Pt 5):1139‐1147. 8176374 10.1099/0022-1317-75-5-1139

[18] Breitkreutz D , Stark HJ , Plein P , Baur M , Fusenig NE . Differential modulation of epidermal keratinization in immortalized (HaCaT) and tumorigenic human skin keratinocytes (HaCaT‐ras) by retinoic acid and extracellular Ca^2+^ . Differentiation. 1993;54(3):201‐217. 7505755 10.1111/j.1432-0436.1993.tb01602.x

[19] Stanley MA , Browne HM , Appleby M , Minson AC . Properties of a non‐tumorigenic human cervical keratinocyte cell line. Int J Cancer. 1989;43(4):672‐676. 2467886 10.1002/ijc.2910430422

[20] Iizuka T , Wakae K , Nakamura M , et al. APOBEC3G is increasingly expressed on the human uterine cervical intraepithelial neoplasia along with disease progression. Am J Reprod Immunol. 2017;78(4):e12703. 10.1111/aji.12703 28590025

[21] Iseki H , Nakachi Y , Hishida T , et al. Combined overexpression of JARID2, PRDM14, ESRRB, and SALL4A dramatically improves efficiency and kinetics of reprogramming to induced pluripotent stem cells. Stem Cells. 2016;34(2):322‐333. 26523946 10.1002/stem.2243

[22] Dallenbach‐Hellweg G . Structural variations of cervical cancer and its precursors under the influence of exogenous hormones. Curr Top Pathol. 1981;70:143‐170. 7297131 10.1007/978-3-642-68185-1_6

[23] Arbeit JM , Howley PM , Hanahan D . Chronic estrogen‐induced cervical and vaginal squamous carcinogenesis in human papillomavirus type 16 transgenic mice. Proc Natl Acad Sci U S A. 1996;93(7):2930‐2935. 8610145 10.1073/pnas.93.7.2930 PMC39737

[24] Chung SH , Lambert PF . Prevention and treatment of cervical cancer in mice using estrogen receptor antagonists. Proc Natl Acad Sci U S A. 2009;106(46):19467‐19472. 19901334 10.1073/pnas.0911436106 PMC2780740

[25] Ali SA , Justilien V , Jamieson L , Murray NR , Fields AP . Protein kinase Ciota drives a NOTCH3‐dependent stem‐like phenotype in mutant KRAS lung adenocarcinoma. Cancer Cell. 2016;29(3):367‐378. 26977885 10.1016/j.ccell.2016.02.012 PMC4795153

[26] Xiao G , Du J , Wu H , et al. Differential inhibition of Sox10 functions by Notch‐Hes pathway. Cell Mol Neurobiol. 2020;40(4):653‐662. 31782037 10.1007/s10571-019-00764-7 PMC11448864

[27] Ojesina AI , Lichtenstein L , Freeman SS , et al. Landscape of genomic alterations in cervical carcinomas. Nature. 2014;506(7488):371‐375. 24390348 10.1038/nature12881 PMC4161954

[28] Cancer Genome Atlas Research N . Comprehensive molecular characterization of urothelial bladder carcinoma. Nature. 2014;507(7492):315‐322. 24476821 10.1038/nature12965 PMC3962515

[29] AbdulMajeed AA , Dalley AJ , Farah CS . Loss of ELF3 immunoexpression is useful for detecting oral squamous cell carcinoma but not for distinguishing between grades of epithelial dysplasia. Ann Diagn Pathol. 2013;17(4):331‐340. 23643910 10.1016/j.anndiagpath.2013.03.003

[30] Ding L , Liu C , Zhou Q , Feng M , Wang J . Association of estradiol and HPV/HPV16 infection with the occurrence of cervical squamous cell carcinoma. Oncol Lett. 2019;17(3):3548‐3554. 30867796 10.3892/ol.2019.10005 PMC6396129

[31] Konishi I , Fujii S , Nonogaki H , Nanbu Y , Iwai T , Mori T . Immunohistochemical analysis of estrogen receptors, progesterone receptors, Ki‐67 antigen, and human papillomavirus DNA in normal and neoplastic epithelium of the uterine cervix. Cancer. 1991;68(6):1340‐1350. 1651807 10.1002/1097-0142(19910915)68:6<1340::aid-cncr2820680626>3.0.co;2-q

[32] Shields TS , Falk RT , Herrero R , et al. A case‐control study of endogenous hormones and cervical cancer. Br J Cancer. 2004;90(1):146‐152. 14710222 10.1038/sj.bjc.6601514 PMC2395325

[33] de The H . Differentiation therapy revisited. Nat Rev Cancer. 2018;18(2):117‐127. 29192213 10.1038/nrc.2017.103

[34] Ablain J , de The H . Retinoic acid signaling in cancer: the parable of acute promyelocytic leukemia. Int J Cancer. 2014;135(10):2262‐2272. 25130873 10.1002/ijc.29081

[35] Helm CW , Lorenz DJ , Meyer NJ , Rising WW , Wulff JL . Retinoids for preventing the progression of cervical intra‐epithelial neoplasia. Cochrane Database Syst Rev. 2013;(6): CD003296. 10.1002/14651858.CD003296.pub3 PMC10468212 23740788

[36] Alvarez RD , Conner MG , Weiss H , et al. The efficacy of 9‐cis‐retinoic acid (aliretinoin) as a chemopreventive agent for cervical dysplasia: results of a randomized double‐blind clinical trial. Cancer Epidemiol Biomarkers Prev. 2003;12(2):114‐119. 12582020

[37] Follen M , Atkinson EN , Schottenfeld D , et al. A randomized clinical trial of 4‐hydroxyphenylretinamide for high‐grade squamous intraepithelial lesions of the cervix. Clin Cancer Res. 2001;7(11):3356‐3365. 11705848

[38] Pashko LL , Rovito RJ , Williams JR , Sobel EL , Schwartz AG . Dehydroepiandrosterone (DHEA) and 3 beta‐methylandrost‐5‐en‐17‐one: inhibitors of 7,12‐dimethylbenz[a]anthracene (DMBA) ‐initiated and 12‐O‐tetradecanoylphorbol‐13‐acetate (TPA) ‐promoted skin papilloma formation in mice. Carcinogenesis. 1984;5(4):463‐466. 6231135 10.1093/carcin/5.4.463

[39] Pashko LL , Hard GC , Rovito RJ , Williams JR , Sobel EL , Schwartz AG . Inhibition of 7,12‐dimethylbenz(a) anthracene‐induced skin papillomas and carcinomas by dehydroepiandrosterone and 3‐beta‐methylandrost‐5‐en‐17‐one in mice. Cancer Res. 1985;45(1):164‐166. 3155493

[40] Suh‐Burgmann E , Sivret J , Duska LR , Del Carmen M , Seiden MV . Long‐term administration of intravaginal dehydroepiandrosterone on regression of low‐grade cervical dysplasia‐a pilot study. Gynecol Obstet Invest. 2003;55(1):25‐31. 12624548 10.1159/000068953

